# 早期肺癌进展趋势的影响因素和CT研判

**DOI:** 10.3779/j.issn.1009-3419.2018.10.11

**Published:** 2018-10-20

**Authors:** 杨波 裘, 锋 毛, 辉 张, 屠阳 申

**Affiliations:** 200030 上海，上海交通大学附属胸科医院/上海市肺部肿瘤临床医学中心 Shanghai Chest Hospital, Shanghai JIaotong University, Department of Thoracic Surgery, Shanghai Lung Tumor Clinical Medical Center, Shanghai 200030, China

**Keywords:** 附壁生长型肺腺癌, 肿瘤进展, 倍增时间, Lepidic predominant adenocarcinoma, Tumor progression, Volume doubling time

## Abstract

**背景与目的:**

不同类型的肺部结节具有不同的体积倍增时间（volume doubling time, VDT）。目前针对不同病理类型早期肺腺癌VDT的研究较少。本研究通过回顾性分析143例早期肺腺癌的影像资料，探讨早期肺腺癌的进展趋势及相关影响因素，为临床制订其随访策略提供参考。

**方法:**

依据2015版世界卫生组织肺肿瘤分类标准和第8版肿瘤肿瘤-淋巴结-转移（tumor-node-metastasis, TNM）分期标准，对143例早期肺腺癌进行分类及分期。参考修正版Schwartz公式计算不同病理类型肺腺癌的VDT。

**结果:**

143例早期肺腺癌中，有50例（34.97%）出现进展，多因素分析显示影响因素包括随访时间、结节大小、病理类型、结节类型和病理分期。附壁生长为主型肺腺癌（lepidic predominant adenocarcinoma, LPA）的VDT为（594±272）d，伴少量附壁生长成分浸润性腺癌的VDT为（520±285）d，完全浸润性腺癌的VDT为（371±183）d，3类进展性早期肺腺癌的VDT有统计学差异（*P*=0.044）。

**结论:**

在早期肺腺癌中，约有35%的肿瘤处于进展阶段，是否含有附壁生长成分是影响肿瘤进展速度的重要因素。

## Abstract (en)

**Background and objective:**

It has been known that the volume doubling time (VDT) of different lung nodule types is different. At present, there is still a lack of studies about the volume doubling time of lung cancer with different pathological types. The purpose of the study is to explore the factors influencing the progression of the early-stage adenocarcinoma, and provide some reference for the follow-up strategy of lung nodules by retrospective analysis of the image data of 143 early-stage adenocarcinoma.

**Methods:**

143 cases of the early adenocarcinoma were classified according to the 2015 World Health Organization Classification of Lung Tumors and the Eighth edition of the tumor-node-metastasis (TNM) classification of lung cancer. The volume doubling time was calculated with reference to the revised Schwartz formula.

**Results:**

Among the 143 cases of the early adenocarcinoma, 50 cases (34.97%) were in progression. By multivarIate analysis, there were several factors associated with the progression of the early adenocarcinoma: the follow-up time, the dimension of nodule, the pathological type, the nodule type and the pathological stage. The VDT of lepidic predominant adenocarcinoma (LPA) is (594±272) d. The VDT of the invasive adenocarcinoma with lepidic part, but not predominant, is (520±285) d. The VDT of the invasive adenocarcinoma without lepidic part is (371±183) d.

**Conclusion:**

About 35% of the early adenocarcinoma is in progress. Whether with the lepidic component is a positive factor to the speed of tumor progression.

肺部结节的体积倍增时间（volume doubling time, VDT）是指结节体积增大一倍所用时间^[1]^。由于不同肺结节的个体差异，其相应VDT亦不尽相同^[2]^。附壁生长（lepidic growth）是指肿瘤细胞沿着已存在的肺泡结构规律生长的一种模式^[3]^。相关临床研究^[4-8]^已经证实，含有不同比例附壁生长成分的肺腺癌患者，其预后状况不同。本研究拟通过回顾性研究本中心143例早期肺腺癌临床病理资料，探讨早期肺腺癌的进展规律及相关影响因素。同时计算进展阶段含不同比例附壁生长成分肺腺癌的VDT值，探讨附壁生长成分对早期肺腺癌进展速度的影响，籍以为肺部结节的随访策略提供依据，并为进一步的研究提供理论基础。

## 1 资料与方法

### 1.1 病例选择

收集2016年1月1日-2017年9月30日在上海市胸科医院肺部肿瘤临床医学中心手术治疗的肺癌患者资料，按照2015版WHO肺肿瘤分类标准（The 2015 World Health Organization Classification of Lung Tumors）对肿瘤病理进行分类^[9]^，依据第8版肿瘤-淋巴结-转移（tumor-node-metastasis, TNM）分期标准对肿瘤术后病理进行分期^[10]^，并根据患者术后病理报告及术前检查结果，选出符合以下标准的病例：①非粘液性腺癌；②肿瘤病理最大径≥6 mm；③术前在上海市胸科医院以常规剂量下（120 KVp, 298 mA-331 mA）胸部多层螺旋计算机断层扫描（multi-detector spiral computer tomography, MDCT）进行随访，随访间隔时间≥3个月且≤5年；④随访期间未行肿瘤相关治疗或穿刺病检；⑤相关影像学检查未发现纵隔淋巴结及远处转移；⑥病理类型除原位癌（adenocarcinoma *in situ*, AIS）、微浸润性腺癌（minimally invasive adenocarcinoma, MIA）外的各类肿瘤，除完整切除肿瘤，均已行系统性淋巴结清扫术（systematic lymph node dissection）。

根据上述标准，本研究共筛选出临床资料完整的肺腺癌143例。结合美国胸科医师协会（American College of Chest PhysicIans, ACCP）和日本临床肿瘤学组对于肺部结节分类（Japan Clinical Oncology Group 0201, JCOG0201）的观点^[11, 12]^，根据结节在CT肺窗下（窗位：-500 HU--700 HU；窗宽：1, 000 HU-2, 000 HU）的表现及结节中实性部分（指结节中无可辨认的支气管、血管结构的部分）最大径与结节最大径的比值，对病灶的类型进行分类：将比值≥0.5的结节称为实性结节（solid nodule, SN），将不含实性部分的结节称为磨玻璃阴影（ground glass opacity, GGO），介于SN和GGO之间的结节称为部分实性结节（part-solid nodule, PSN）。

### 1.2 影像研判

所有病例的病理标本均经2位高年资病理医生阅片审核。根据2015版WHO肺肿瘤分类标准^[9]^、是否含有附壁生长成分及其是否为肺腺癌的主要生长方式，对所有病例的病理按如下进行分类：AIS、MIA、附壁生长为主型浸润性腺癌（lepidic predominant invasive adenocarcinoma, LPIA）、伴少量附壁生长成分的浸润性腺癌、无附壁生长成分的浸润性腺癌。重新调阅随访期间的CT资料，对不同时间各结节在横断面下的最长径和同一层面下的最大垂直径分别进行测量。VDT的计算参考Usuda等所使用的修正版Schwartz公式^[1]^：VDT=(t×log^2^)/[log(V1/V0)]，其中V0和V1分别指结节首次随访和末次随访体积，t指首末两次随访所间隔时间，V=π/6×ab²（a、b分别指最长径和最大垂直径）。计算首末两次随访之间结节的最长径（或最大垂直径）差值^[13]^，若该值≥2 mm，则将该结节归入进展性肺腺癌组（increasing group）；若该值< 2 mm，则将该结节归入无进展性肺腺癌组（not increasing group）。

### 1.3 统计学方法

采用SPSS Statistics 24.0软件对数据进行统计学分析。计量资料以均数±标准差（Mean±SD）表示，组间比较采用*Mann-Whitney U*或*Kruskal Wallis ANOVA*检验；计数资料比较采用*χ²*检验或*Fisher’s*精确检验；多因素分析采用*Log rank*检验；以*P* < 0.05为具有显著统计学差异。

## 2 结果

### 2.1 临床病例特征

本研究共纳入2016年1月1日-2017年9月30日在上海市胸科医院肺部肿瘤临床医学中心行手术治疗的肺腺癌143例，随访时间（14±11）个月。病例临床特征包括：年龄、性别、肿瘤所在位置、病理类型、结节类型、pTNM分期、是否进展（表 1）。在所有病例中，患者主要由女性构成，少数为男性。肿瘤以右肺上叶最多见，随后依次为左肺上叶、右肺下叶、左肺下叶，右肺中叶最少。结节类型以GGO最多见，其次为PSN，SN最少。分期Ia_1_期最多，Ia_2_和0期（AIS）其次，Ia_3_期和Ib期最少。约有2/3结节未出现进展，约有1/3结节发生进展。

**1:** 各病灶的临床特征 Clinical characteristics of the lesions

Clinical characteristics	Data
Total	143 (100.00%)
Age (Mean±SD, yr)	61±9
Gender	
Female	100 (69.93%)
Male	43 (30.07%)
Location	
Right upper lobe	53 (37.06%)
Right middle lobe	7 (4.90%)
Right lower lobe	28 (19.58%)
Left upper lobe	33 (23.08%)
Left lower lobe	22 (15.38%)
Pathological types	
AIS	22 (15.38%)
MIA	44 (30.77%)
LPIA	17 (11.89%)
With lepidic but not predominant	32 (22.38%)
Without lepidic	28 (19.58%)
Nodule types	
GGO	63 (44.06%)
PSN	47 (32.87%)
SN	33 (23.08%)
Pathological stage	
0	22 (15.38%)
Ia_1_	71 (49.65%)
Ia_2_	40 (27.97%)
Ia_3_	8 (5.59%)
Ib	2 (1.40%)
Being increasing	
Yes	50 (34.97%)
No	93 (65.03%)
AIS: adenocarcinoma in situ; MIA: minimally invasive adenocarcinoma; LPIA: lepidic predominant invasive adenocarcinoma; GGO: ground-glass opacity; PSN: part-solid nodule; SN: solid nodule.	

### 2.2 进展趋势分析

分析进展组和无进展组的临床病理资料，发现两组在年龄（*P*=0.065）、性别构成（*P*=0.129）、肿瘤位置（*P*=0.819）方面无统计学差异，而在随访时间（*P* < 0.001）、结节大小（*P* < 0.001）、结节类型（*P* < 0.001）、病理类型（*P* < 0.001）、pTNM分期上（*P*=0.001）有统计学差异（表 2）。由表 2可见，进展组的随访时间更长、肿瘤最大径值更大。进一步绘制关于随访时间（图 1）和结节大小（图 2）的受试者工作特征曲线（receiver operating characteristic curve, ROC curve），可以发现：当随访时间为15.65个月、结节大小为12.15 mm时，特异性（specificity）和敏感性（sensitivity）最佳。对3种结节类型进行两两比较发现：GGO进展可能性最低，PSN其次，SN最高。对5种病理类型进行两两比较发现：AIS、MIA和LPIA进展可能性低，含少量附壁生长成分的肺腺癌、无附壁生长成分的肺腺癌进展可能性高；AIS、MIA和LPIA之间，LPIA和含少量附壁生长成分的肺腺癌之间，含少量附壁生长成分的肺腺癌和无附壁生长成分的肺腺癌之间的进展可能性无统计学差异。同时，对不同pTNM分期的肺腺癌进行两两比较发现：0期、Ia_1_期和Ia_2_期肺腺癌进展可能性较低，Ia_3_期进展可能性高。进展性肺腺癌组VDT为（488±261）d。

**2:** 无进展组与进展组的临床及病理特征对比 Clinical and pathological characteristics between the groups whether being increasing

Characteristics	Not increasing group (*n*=93)	Increasing group (*n*=50)	*P*
Age (Mean±SD, yr)	60±10	62±9	0.065
Follow-up time (Mean±SD, mo)	12±11	18±11	< 0.001
Nodule size at last (Mean±SD, mm)	11±4	16±8	< 0.001
Sex			0.129
Female	69	31	
Male	24	19	
Location			0.819
Right upper lobe	32	21	
Right middle lobe	4	3	
Right lower lobe	18	10	
Left upper lobe	23	10	
Left lower lobe	16	6	
VDT (Mean±SD, d)	/	488±261	/
Pathological types			< 0.001
AIS	19	3	
MIA	36	8	
LPIA	13	4	
With lepidic but not predominant	15	17	
Without lepidic	10	18	
Nodule types			< 0.001
GGO	53	10	
PSN	28	19	
SN	12	21	
Pathological stage			0.001
0	19	3	
Ia_1_	50	21	
Ia_2_	23	17	
Ia_3_	1	7	
Ib	0	2	
AIS: adenocarcinoma in situ; MIA: minimally invasive adenocarcinoma; LPIA: lepidic predominant invasive adenocarcinoma; VDT: volume doubling time.			

**1:**
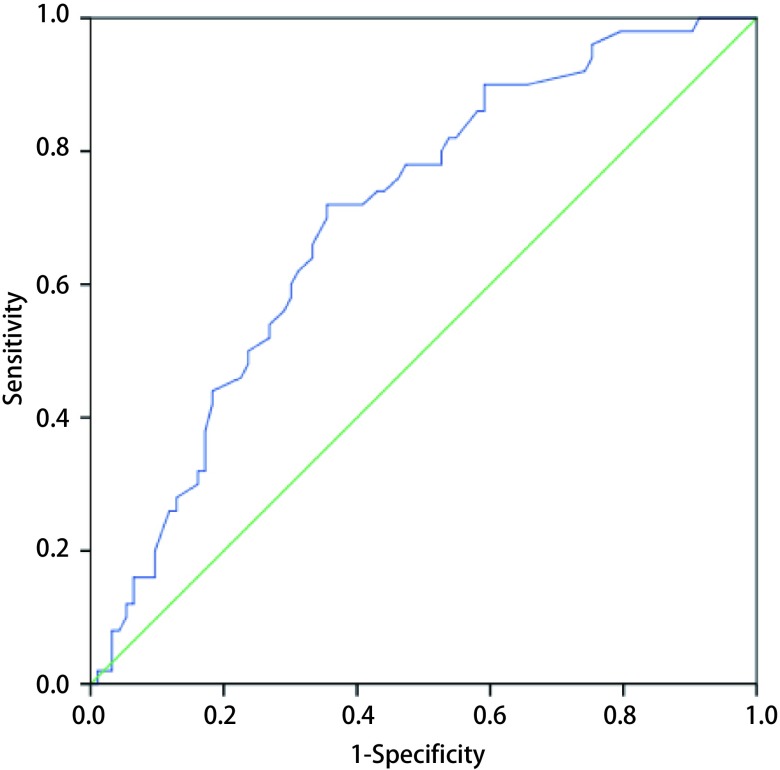
随访时间对判断肺腺癌进展的影响 The receiver operating curve (ROC) of the follow-up time to the increasing of adenocarcinoma (AUC=0.700, 95%CI: 0.613-0.787, *P* < 0.001)

**2:**
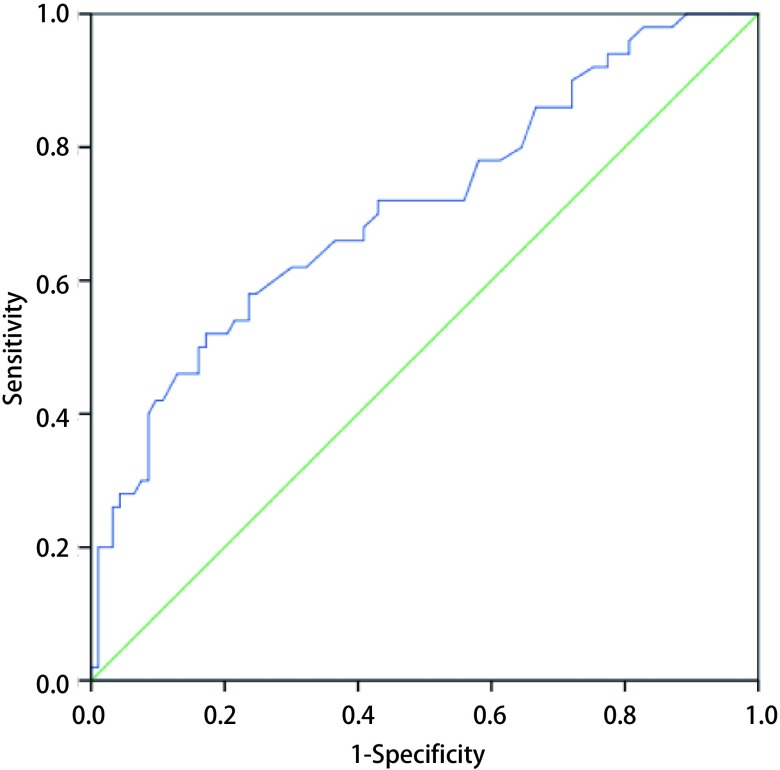
结节大小对判断肺腺癌进展的影响 The receiver operating curve (ROC) of the nodule size to the increasing of adenocarcinoma (AUC=0.710, 95%CI: 0.619-0.801, *P* < 0.001)

### 2.3 附壁生长的影响

依据不同病理类型对5类进展性肺腺癌的随访时间、结节大小和VDT进行分析（表 3），发现：VDT无统计学差异（*P*=0.056），随访时间（*P*=0.042）和结节大小（*P*=0.047）存在统计学差异。为减少随访时间和结节大小两因素对早期肺腺癌进展速度的干扰，故重新将进展性早期肺腺癌分为3类：LPA、含少量附壁生长成分的早期肺腺癌和无附壁生长成分的早期肺腺癌。发现3类进展性早期肺腺癌的VDT有统计学差异（*P*=0.044），随访时间（*P*=0.091）和结节大小（*P*=0.086）无统计学差异。对3类进展性早期肺腺癌的VDT进行两两比较发现：差异主要发生在LPA（594±272）d和无附壁生长成分的早期肺腺癌（371±183）d之间，含少量附壁生长成分的早期肺腺癌（520±285）d和其余两组无明显差异（表 4）。

**3:** 不同病理类型进展性肺腺癌的VDT（5分类）(Mean±SD) The VDT of the increasing group among different pathological types (5 types) (Mean±SD)

Characteristics	AIS (*n*=3)	MIA (*n*=8)	LPIA (*n*=4)	With lepidic but not predominant (*n*=17)	Without lepidic (*n*=18)	*P*
Follow-up time (mo)	22±12	16±8	36±12	18±11	14±8	0.042
Nodule size at last (mm)	8±1	15±5	12±2	18±8	17±9	0.047
VDT (d)	396±196	595±282	741±257	520±285	371±183	0.056

**4:** 不同病理类型进展性肺腺癌的VDT（3分类）(Mean±SD) The VDT of the increasing group among different pathological types (3 types) (Mean±SD)

Characteristics	LPA (*n*=15)	With lepidic but not predominant (*n*=17)	Without lepidic (*n*=18)	*P*
Follow-up time (mo)	23±13	18±11	14±8	0.091
Nodule size at last (mm)	13±4	18±8	17±9	0.086
VDT (d)	594±272	520±285	371±183	0.044
LPA: lepidic predominant adenocarcinoma.				

## 3 讨论

自2011年2月，国际肺癌研究学会（The International AssocIation for the Study of Cancer, IaSLC）、美国胸科学会（American Thoracic Society, ATS）和欧洲呼吸协会（European Respiratory Society, ERS）联合发表关于肺腺癌的国际多学科分类以来^[12]^，国内外已发表多篇针对不同病理类型肺腺癌预后状况的随访研究^[4-8]^。这些研究发现：AIS、MIA的预后状况最佳，LPIA其次，其余病理类型的肺腺癌较差。而在临床上，随着CT逐渐成为肺癌筛查的首选方式以来，越来越多的早期肺腺癌得以被及时发现^[2]^。正确评估0期、Ⅰ期（包括Ia各期和Ib期）肺腺癌的进展状况，对早期肺腺癌手术方案的选择以及肺部结节随访策略的制定，都有重要的意义。

在2017版Fleischner协会肺部结节处理指南（Guidelines for Management of Incidental Pulmonary Nodules Detected on CT Images: From the Fleischner Society 2017）中^[2]^，研究者认为：肺部结节的随访间隔时间和随访周期，应当根据不同类型结节的VDT而定。对那些VDT较长的结节，除适当延长随访周期外，还应谨慎选择手术介入时机，避免因过早介入而造成过度诊断和治疗^[13, 14]^。然而，不同研究由于病例来源、临床信息、结节类型、结节大小和病理类型等方面的差异，得出的VDT结果并不相同。Hasegawa等^[15]^对61例各类肺部结节CT资料的分析发现：GGO、PSN、SN的VDT分别为813 d、457 d和149 d。Chang等^[13]^分析122例GGO影像学资料，发现其中12例（9.84%）出现增大，VDT为769 d。此外，应当注意到，结节在进展时，除了大小的改变，还伴随有密度的改变。Takashima等^[16]^分析73例肺部结节随访图像，认为在GGO的进展中，通常先出现结节长度的增加，随后出现少量实性成分，最后变成纯实性结节（pure solid nodule）。Yankelevitz等^[17]^分析84例GGO的临床资料发现：22例（26.19%）出现实性成分的增加，从GGO演变为PSN的平均时间为25个月。Kakinuma等^[18]^则通过观察1, 046例GGO随访过程中的实性成分发现：有13例演变为肺窗下的PSN，54例演变为纵隔窗下的PSN，平均时间分别为2.1年和3.8年。在上述研究中，由于不同结节之间的病理类型不同，而不同病理类型所对应的患者预后差异较大，因此，根据结节类型计算出的VDT及结节类型变化时间，可能并不完全适用于临床上肺部结节的分析。在本研究中，研究者通过对进展组和无进展组肺腺癌之间差异的分析发现：与无进展组肺腺癌相比，进展组的结节随访时间更长、结节长径更大。借助ROC曲线及相应计算结果，我们认为：当结节持续随访时间约16个月，或者结节大小约12 mm时，需与之前的CT进行对比，以便及时发现有进展的结节。

Song等^[19]^根据结节的病理类型，分别计算不同病理类型肺腺癌的VDT，16例AIS约为1, 240.3 d，3例MIA约为1, 328.3 d，7例浸润性腺癌（invasive adenocarcinoma, IA）约为941.5 d（*P*=0.382）。该研究未对结节长度的差异进行区分，而相关研究已经证实：结节大小差异 < 1.73 mm时，差异由测量误差造成的可能性较大^[20]^。因此，Kakinuma等^[18]^认为实际AIS、MIA或者IA的进展速度应更快。在本研究中，根据肺腺癌中附壁样生长成分多少，计算进展组各类肺腺癌的VDT：LPA为（594±272）d，含少量附壁样生长成分肺腺癌为（520±285）d，无附壁样成分肺腺癌为（371±183）d（*P*=0.044），差异主要出现于LPA与无附壁样成分肺腺癌之间。这一结果说明：可能肺腺癌的进展并非匀速，当肿瘤的生长模式发生改变，其生长速度亦随之改变。

针对“肺腺癌如何演变，是否沿着某一固定生长模式进展？”等问题，不同研究者看法各异。Yasushi认为在肺腺癌演变过程中，一部分肺腺癌是沿着“不典型腺瘤样增生（atypical adenomatous hyperplasIa, AAH）、AIS、MIA、IA”的线性进展模式（linear progression schema）演变，有一部分可能会在中间某一阶段减速、加速或停止，还有一部分可能转变为其他模式^[21]^。Lindell等^[22]^回顾分析18例肺癌的影像学资料（每例至少4次），认为在不同长度时，肿瘤的进展速度都不一样，根据前一阶段的进展速度推断肿瘤的后续进展速度是不合理的。在本研究中，通过对不同病理类型肺腺癌进展可能性的分析及相应进展速度的计算，可以发现：在肺腺癌以附壁生长为主要生长模式时，肿瘤的进展可能性较低，随着附壁成分的减少，肿瘤进展的可能性逐渐增加，与LPA相比，无附壁样生长成分肺腺癌的进展速度较快。如果肺腺癌沿着线性进展模式演变，考虑到肿瘤进展早期的速度较慢，当肿瘤进展至无附壁样生长成分阶段时，其实际进展速度应当更快。

综上所述，在0期-Ⅰ期的早期肺腺癌中，约有35%处于进展阶段，影响肿瘤进展趋势的因素包括：随访时间、结节大小、结节类型、病理类型和pTNM分期。在肺部结节的随访过程中，若结节持续存在达16个月，或者结节长度为12 mm时，需及时复查CT并与前期图像进行对比。附壁生长作为早期肺腺癌生长的主要模式，进展速度明显较低，随着附壁成分的减少，肿瘤的进展速度将明显加快。考虑到和含附壁样成分肺腺癌相比，无附壁样生长成分肺腺癌在预后上较差，若结节在随访期间出现较前更快的进展速度，无论结节大小，都应及时手术介入。
